# Have guidelines been oblivious to the obvious? Unmasking the positives from the COVID-19 pandemic

**DOI:** 10.1038/s41533-021-00224-0

**Published:** 2021-02-26

**Authors:** K. Vishak Acharya, B. Unnikrishnan, K. Sindhu, S. Deepthi

**Affiliations:** 1 Department of Pulmonary Medicine, Kasturba Medical College, Mangalore, MAHE, Manipal, India; 2 Department of Preventive & Community Medicine, Kasturba Medical College, Mangalore, MAHE, Manipal, India

**Keywords:** Diseases, Chronic obstructive pulmonary disease

Global Initiative for Chronic Obstructive Lung Diseases (GOLD)^1^ guidelines for chronic obstructive pulmonary disease (COPD) and Global Initiative for Asthma (GINA)^2^ for asthma are the two most seminal guidelines practiced by physicians worldwide providing a benchmark in care. The prevailing practices adopted during the coronavirus disease 2019 (COVID-19) pandemic pertaining to hygiene, personal protection strategies, and quarantine measures have opened up unforeseen possibilities of these basic measures being one of the key factors that could reduce exacerbation rates in asthma and COPD. This pandemic may just provide the right framework for these comprehensive guidelines to be revisited to review their present recommendations.

During the COVID-19 pandemic, there has been a lot of focus on its impact on those with comorbidities in whom the outcome, in terms of hospitalizations and mortality, has been higher. However, surprisingly and contrary to the general perception, overall prevalence of COPD and bronchial asthma patients affected by COVID-19 has been quite low as compared to the global burden of these diseases^3–6^. Several theories have been proposed to explain this paradox. These range from protective effects of inhaled corticosteroids, a different immune response in patients with COPD, under diagnosis of chronic respiratory diseases (CRDs), and possible benefits of hygiene and isolation measures^6^.

By contrast, the disease severity and overall outcome in COVID-19 patients with CRD has been more severe. Reports from across the globe state CRDs as among the most frequent comorbidities associated with severe COVID illnesses requiring intensive care unit admissions, need for mechanical ventilation, and deaths^7,8^. This intriguing association of CRDs in those with mild COVID compared to those with severe illness is not clearly understood.

A common clinical observation shared by physicians worldwide has been a significant reduction of acute exacerbation of COPD (AECB) and asthma in the last quarter of 2020. The impact of the pandemic has resulted in appreciably better air quality. These are likely due to reduced pollution and extreme measures practiced globally to avoid respiratory transmission of infections. Concerns about access to health care and awareness among patients that optimized treatment of their lung disease lowers their risk of COVID-19 complications probably are factors leading to good adherence to prescribed medications. It is plausible that an interplay of these factors may have resulted in reduced AECB and exacerbation of asthma, although this causal relationship needs to be confirmed.

Data emerging from systematic review and meta-analysis have demonstrated lower risk of infection for physical distancing of 1 m as was with the use of face masks. Added benefits are likely with physical distances of ≥2 m based on modeling by meta-regression analysis^9^. Such studies could also encourage better adherence to the use of face masks and respirators in public and health-care settings. Robust randomized trials are, however, needed to gauge and substantiate the evidence for these interventions. Systematic appraisal of currently available evidence might pave the way for interim guidance on effectiveness of these basic and common interventions in combating the immediate threat of COVID-19 infections^9^.

The landmark guidelines GOLD and GINA have been surprisingly silent on basic sanitary and respiratory hygiene measures and have overlooked the role of masks in certain specific settings. Recommending the use of masks for stratified high-risk group of CRD patients in certain settings such as mass gathering may be a judicious and far-sighted move. Weighing the scientific uncertainty and the contextual considerations, there is a need for a more nuanced view by members of the task force formulating the guidelines. It would only be prudent to include these basic lifestyle modifications and preventive measures among the non-pharmacological interventions for COPD and asthma in the guidelines. GOLD and GINA should also consider updating their guidelines with practical recommendations for personal protection equipment and other hygiene measures for safe spirometry procedures. Presently, this phenomenon is largely based on non-scientific observations from the real-world clinical practice. Data from randomized controlled trials and other scientific studies are needed to validate these preliminary observations.

Exacerbations are the primary factors that modify the long-term disease progression in these CRDs. Therefore, these above-mentioned basic but feasible and universally adaptable measures have a promising role along with pharmacotherapy in potentially reducing exacerbation. As a result, these measures could modify the overall disease outcome in CRDs.

## Data Availability

No datasets were generated or analyzed during the current study.

## References

[1] Global Initiative for Chronic Obstructive Lung Disease (GOLD). Global strategy for the diagnosis, management and prevention of COPD. http://goldcopd.org/gold-2020-globalstrategy-diagnosis-management-prevention-copd (2020).

[2] Global Initiative for Asthma (GINA). Global strategy for the diagnosis and prevention. Global Initiative for Asthma (updated 2020). http://ginasthma.org/wpcontent/uploads/2020/04/GINA-2020-full-report_-final (2020).

[3] Emami A, Javanmardi F, Pirbonyeh N, Akbari A (2020). Prevalence of underlying diseases in hospitalized patients with COVID-19: a systematic review and meta-analysis. Arch. Acad. Emerg. Med..

[4] Grasselli G (2020). Baseline characteristics and outcomes of 1591 patients infected with SARS-CoV-2 admitted to ICUs of the Lombardy Region, Italy. JAMA.

[5] Goyal P (2020). Clinical characteristics of Covid-19 in New York City. N. Engl. J. Med..

[6] Halpin DMG, Faner R, Sibila O, Badia JR, Agusti A (2020). Do chronic respiratory diseases or their treatment affect the risk of SARS-CoV-2 infection?. Lancet Respir. Med..

[7] Khaltaev N (2017). GARD, a new way to battle with chronic respiratory diseases, from disease oriented programmes to global partnership. J. Thorac. Dis..

[8] Guan WJ (2020). Comorbidity and its impact on 1590 patients with COVID-19 in China: a nationwide analysis. Eur. Respir. J..

[9] Chu DK (2020). Physical distancing, face masks, and eye protection to prevent person-to-person transmission of SARS-CoV-2 and COVID-19: a systematic review and meta-analysis. Lancet.

